# Stress and burnout among critical care fellows: preliminary evaluation of an educational intervention

**DOI:** 10.3402/meo.v20.27840

**Published:** 2015-07-23

**Authors:** Kianoush Kashani, Perliveh Carrera, Alice Gallo De Moraes, Amit Sood, James A. Onigkeit, Kannan Ramar

**Affiliations:** 1 Division of Pulmonary and Critical Care, Department of Medicine, Mayo Clinic, Rochester, MN, USA; 2 Division of Nephrology and Hypertension, Department of Medicine, Mayo Clinic, Rochester, MN, USA; 3 Division of General Internal Medicine, Department of Medicine, Mayo Clinic, Rochester, MN, USA; 4 Department of Anesthesiology, Mayo Clinic, Rochester, MN, USA

**Keywords:** survey research, critical care, mental health, program evaluation, medical education, burnout

## Abstract

**Background:**

Despite a demanding work environment, information on stress and burnout of critical care fellows is limited.

**Objectives:**

To assess 1) levels of burnout, perceived stress, and quality of life in critical care fellows, and 2) the impact of a brief stress management training on these outcomes.

**Methods:**

In a tertiary care academic medical center, 58 critical care fellows of varying subspecialties and training levels were surveyed to assess baseline levels of stress and burnout. Twenty-one of the 58 critical care fellows who were in the first year of training at the time of this initial survey participated in a pre-test and 1-year post-test to determine the effects of a brief, 90-min stress management intervention.

**Results:**

Based on responses (*n*=58) to the abbreviated Maslach Burnout Inventory, reported burnout was significantly lower in Asian fellows (*p*=0.04) and substantially higher among graduating fellows (versus new and transitioning fellows) (*p*=0.02). Among the intervention cohort, burnout did not significantly improve – though two-thirds of fellows reported using the interventional techniques to deal with stressful situations. Fellows who participated in the intervention rated the effectiveness of the course as 4 (IQR=3.75–5) using the 5-point Likert scale.

**Conclusions:**

In comparison with the new and transitioning trainees, burnout was highest among graduating critical care fellows. Although no significant improvements were found in first-year fellows’ burnout scores following the single, 90-min training intervention, participants felt the training did provide them with tools to apply during stressful situations.

Multiple studies have shown that distress symptoms, such as depersonalization, emotional exhaustion, and sense of personal under-accomplishment, are common among medical students, residents, and staff physicians (1–5). Burnout is a psychological syndrome arising in response to chronic emotional and interpersonal stressors on the job (6). In a study by Embriaco et al., 50% of intensivists exhibited burnout, and those with higher levels of burnout reported conflicts with coworkers (7). Moreover, stress symptoms and depression among doctors are reported to be associated with lowered standards of patient care, medical mistakes, and sometimes even leading to patient death (8–10). Patients treated by physicians experiencing burnout are also less satisfied with the care they receive. Also, residents reporting burnout are more likely to commit medical errors (11, 12). Given the negative impacts of stress and burnout to a person's well-being, interventions such as resilience training have been tested in controlled studies with promising results (13, 14).

Although they are exposed to a high-stress environment of the intensive care unit, studies focusing on stress and burnout among fellows in training are scarce (4, 5, 10, 15). A study by Mougalian showed that more than half of oncology fellows reported burnout in at least one domain of the Maslach Burnout Inventory (MBI) (16). A Dutch study noted general surgery and obstetrics-gynecology residents had the lowest burnout (12 and 15%, respectively), while burnout among internal medicine residents was 24% (17). As a majority of critical care fellows have internal medicine backgrounds, this may suggest an increased tendency for experiencing burnout.

We were unable to identify any interventional studies conducted to date on the fellows to address their burnout and stress. Sood and associates reported several studies showing short interventions, including a 90-min session, were able to decrease stress and burnout in different populations of health care providers (18–20). In this cross-sectional study, then, we explore the impact on critical care fellows of a single 90-min stress management training session adapted from the Stress Management and Resiliency Training (SMART) program developed at Mayo Clinic (19).

## Methods

After obtaining the institutional review board (IRB) approval (IRB# 13-009252), a 2013 survey was obtained from all adult critical care fellows enrolled in the Pulmonary and Critical Care Medicine (PCCM), Critical Care Internal Medicine (CCM), Critical Care Anesthesia, and Neurology Critical Care (NCC) training fellowship programs at Mayo Clinic (Rochester, MN). Fifty-eight of the 62 critical care fellows (93.5%) in the first, second, and third years of training agreed to participate in the initial survey. In the second phase of this study, 21 of the 58 critical care fellows who were in the first year of training at the time of this initial survey participated in a pre-test and 1-year post-test to determine the effects of a brief, 90-min stress management intervention. Out of these 21, 18 responded to the survey (86% response rate).

Fellows were stratified based on their sex, age, and type, stage, and current year of fellowship training. The stages of fellowship were categorized as 1) graduating fellows in their last year of training; 2) transitional fellows about to be promoted to the next year of training; and 3) new fellows just joining their program.

In addition to basic demographic data, the survey instrument contained the Gratitude Questionnaire-Six Item Form (GQ-6) (21), Satisfaction with Life Scale (SWLS) (22), Subjective Happiness Scale (SHS) (23), abbreviated MBI (24) scale, and the 14-item Perceived Stress Scale (PSS-14) (25). The full 22-item MBI was abbreviated into single-item measures of emotional exhaustion and depersonalization (aMBI) (26). The confidential, ~10-min questionnaire was administered electronically using REDCap Software (Version 1.3.10-^©^2013 Vanderbilt University) – along with weekly reminders.

After this initial survey, the 90-min stress management intervention – adapted from the previously-piloted SMART program at Mayo Clinic (18, 19) – was offered to the group of 21 first-year critical care fellows. The program consisted of a presentation and handouts addressing the causes of stress, and introduced ways of framing one's mindset by attention training (i.e., focusing on the novel and shifting one's attention from inward to outward). Finally, fellows were provided with structured relaxation training by utilizing paced breathing meditation and deep diaphragmatic breathing. We surveyed all 21 fellows who participated in the intervention after 1 year to assess changes in their perceived stress, burnout, and overall quality of life.

The results for continuous variables are reported using the: 1) mean and standard deviation (SD) and 2) median and inter-quartile ranges (IQR); categorical data are presented using counts (%). Mean differences were compared using ANOVA, *t*-test, and Wilcoxon rank-sum test. Bonferroni adjustments were made for multiple comparisons. The Wilcoxon signed rank test was utilized to analyze the pre- and post-intervention burnout scores. A *p* value of ≤ 0.05 was considered statistically significant. Data analyses were done using JMP software (Version 9.01; SAS Institute Inc., Cary, NC).

## Results

In the baseline study, 58 of 62 (93.5%) fellows consented to participate; of these, the survey response rate was 100% – with all 58 fellows providing complete data. For the pre/post survey of first-year fellows, 86% of the 21 participants completed both questionnaires. Table 1 shows the socio-demographic characteristics of the 58 fellows comprising the initial sample.

**Table 1:** Baseline characteristics of participating fellows

Characteristic	Frequency (%)
	*N*=58
Male	35 (60)
Age	
25–29	8 (14)
30–34	37 (64)
35–39	10 (17)
40–44	2 (3)
45–50	1 (2)
Race/ethnicity	
Hispanic or Latino	4 (7)
Asian, Middle Eastern, Indian	19 (33)
Black or African American	2 (3)
White	33 (57)
Fellowship type	
CCM	30 (52)
PCCM	20 (34)
CCA	4 (7)
NCC	4 (7)
Fellowship stage	
New	22 (38)
Transition	16 (28)
Graduating	20 (34)

Critical Care Medicine (CCM); Pulmonary and Critical Care Medicine (PCCM); Critical Care Anesthesiology (CCA); Neurology Critical Care (NCC).

As shown in Table 2, the average score on the PSS-14 was 21.4±6.7 (mean±SD), whereas the median GQ-6 score was 38.5 (36–41), the median SWLS score was 28 (24–30), the median SHS score was 22.5 (18–25), and the median aMBI score was 2 (1–6).

**Table 2:** Median PSS-14, GQ-6, SWLS, SHS, and aMBI score, based on demographic variables in the cohort of 58 fellows who participated in the original survey

Variable	Subgroup	PSS-14 (median)	GQ-6 (median)	SWLS (median)	SHS (median)	aMBI (median)
Gender	Male	21	38	27	22	2
	Female	21	41	29	25	2
Age	25–29	8	40	30	25	6
	30–34	16.5	39.5	29	25	0.5
	35–39	22.5	39.5	22.5	22.5	3.5
	40–44	21	38	28	22	2
	45–50	23	38	28	20	1.5
Race/ethnicity	Hispanic or Latino	21	39	29.5	25	2
	Asian, Middle Eastern, Indian	21	38	26	22	1*
	Black or African American	21	41.5	27	21	5.5
	White	21	38	28	22	3
Fellowship type	CCM	23	38.5	26	22	2.5
	PCCM	19	38.5	29*	23.5	2
	CCA	21.5	39	28	21.5	5.5
	NCC	21.5	37	25	16.5	2
Fellowship stage	New	19.5	38.5	28	23.5	1
	Transition	21	38.5	29	24	2.5
	Graduating	23.5	38.5	24.5	22	4*
SMART Program	Pre-intervention	22	38	27	22	2
	Post-intervention	33*	30*	27	19*	3

* Statistically significant (*p*<0.05) different in each demographic category.

In a linear regression analyses (not shown), we examined the unique predictive effects of sex, age, race/ethnicity, and type and stage of fellowship on fellows’ scores on each of the aforementioned variables. The lowest mean SWLS score was in the 35–39 age group (*p*=0.03). Fellows originating from Asia (immigrants to the United States) had significantly lower aMBI scores than did Caucasians (*p=*0.04). In comparison to their CCM counterparts, PCCM fellows reported significantly higher SWLS scores (*p=*0.01). No differences in the scores were noted by fellowship year. However, graduating fellows had significantly higher MBI scores in comparison with the new and transitioning fellows (*p*=0.02).

Among the 18 fellows who completed the pre-/post-test survey, no significant changes were noted in mean satisfaction with life scale (SWLS) or aMBI scores. Interestingly, mean PSS-14 scores were significantly higher at follow-up (*p*≤0.0001), whereas both GQ-6 (*p*≤0.0001) and SHS (*p=*0.04) scores were significantly lower 1 year after the resilience training. Despite no documented improvements in fellows’ self-reported levels of burnout, 67% expressed a strengthened ability to deal with stressful situations, and 61% reported using the techniques after 1 year.

## Discussion

In our cross-sectional study, we found differences in stress levels, gratitude, happiness, satisfaction with life, and burnout based on age, sex, and type and stage of fellowship – but not fellowship subspecialty. Also, graduating fellows reported significantly higher burnout, and Asian international medical graduates (IMGs) expressed significantly lower levels of burnout. In our pre-/post-intervention study, we found no significant improvement in the fellows’ burnout scores – though the training itself was felt to be beneficial.

Our results are different from those reported for other medical trainees and practicing physicians (4, 5, 10, 27). Observed aMBI scores were lower than for other U.S. internal medicine residents, and we noted a curious ethnic variation among Asian IMG critical care fellows – who reported lower levels of burnout. Whether cultural, religious, or spiritual factors played a role in this apparent resilience is not entirely clear.

Mean PSS-14 scores in our study were also lower than those seen in community samples (21.0 vs. 23.5 and 25.6 for males and females, respectively) (28), which could be due to fellows’ self-selection into advanced critical care training. That graduating fellows were found to have higher burnout scores than either new or transitioning fellows might be related to the accumulated length of training in a stressful environment and/or the uncertainty of finding a new job.

In terms of the educational intervention aimed at reducing stress and burnout, participants after 1 year demonstrated significantly higher stress levels and lower gratitude and happiness measures, whereas burnout and satisfaction remained unchanged. Aside from the brief and singular nature of the program itself, the survey timing may have played a role for graduating fellows – who were actively engaged in job seeking, while others were applying for other subspecialty fellowships and further training. Furthermore, as mentioned, the intervention program was very brief (one 90-min session), without any reinforcement or follow-up for 1 year. Our results were not as impressive as previous studies of the original SMART intervention. Perhaps our adaptation was less intensive, had fewer follow-up sessions, and greater one-on-one interaction (18, 19).

There are several limitations to our study. First, the small sample size and singular study setting reduce the rigor and generalizability of our findings. Second, the lack of a comparison or control group tempers any causal attributions of the intervention program. Third, although fellows were assured that their responses were confidential; it is unclear how free they felt to share their opinions unreservedly and honestly. Last, numerous potential confounds exist for which we did not have measures (e.g., social support) that could help explain the observed differences among fellows’ ethnic backgrounds (for example).

## Conclusion

Although critical care fellows were clearly not without stressors, they reported comparatively less baseline stress and burnout and higher levels of gratitude, happiness, and satisfaction with life than other medicine training programs (though stress and burnout levels did increase as fellows moved closer to graduation). Although the brief stress management training did not improve stress, gratitude, or satisfaction (18), fellows subjectively felt some merit to the program. Future studies of more intensive interventions are needed to address stress and burnout among advanced trainees (7, 29).

## References

[1] Collier VU, McCue JD, Markus A, Smith L (2002). Stress in medical residency: status quo after a decade of reform?. Ann Intern Med.

[2] Thomas NK (2004). Resident burnout. JAMA.

[3] Shanafelt TD, Sloan JA, Habermann TM (2003). The well-being of physicians. Am J Med.

[4] Shanafelt TD, Bradley KA, Wipf JE, Back AL (2002). Burnout and self-reported patient care in an internal medicine residency program. Ann Int Med.

[5] Dyrbye LN, West CP, Satele D, Boone S, Tan L, Sloan J (2014). Burnout among U.S. medical students, residents, and early career physicians relative to the general U.S. population. Acad Med.

[6] Maslach C, Schaufeli WB, Leiter MP (2001). Job burnout. Ann Rev Psychol.

[7] Embriaco N, Azoulay E, Barrau K, Kentish N, Pochard F, Loundou A (2007). High level of burnout in intensivists: prevalence and associated factors. Am J Respir Crit Care Med.

[8] Firth-Cozens J, Greenhalgh J (1997). Doctors’ perceptions of the links between stress and lowered clinical care. Soc Sci Med.

[9] Fahrenkopf AM, Sectish TC, Barger LK, Sharek PJ, Lewin D, Chiang VW (2008). Rates of medication errors among depressed and burnt out residents: prospective cohort study. BMJ.

[10] West CP, Tan AD, Habermann TM, Sloan JA, Shanafelt TD (2009). Association of resident fatigue and distress with perceived medical errors. JAMA.

[11] Haas JS, Cook EF, Puopolo AL, Burstin HR, Cleary PD, Brennan TA (2000). Is the professional satisfaction of general internists associated with patient satisfaction?. J Gen Int Med.

[12] Prins JT, van der Heijden FMMA, Hoekstra-Weebers JEHM, Bakker AB, van de Wiel HBM, Jacobs B (2009). Burnout, engagement and resident physicians’ self-reported errors. Psychol Health Med.

[13] Schiraldi GR, Jackson TK, Brown SL, Jordan JB (2010). Resilience training for functioning adults: program description and preliminary findings from a pilot investigation. Int J Emerg Ment Health.

[14] Burton NW, Pakenham KI, Brown WJ (2010). Feasibility and effectiveness of psychosocial resilience training: a pilot study of the READY program. Psychol Health Med.

[15] Prins JT, Gazendam-Donofrio SM, Tubben BJ, Van Der Heijden FMMA, Van De Wiel HBM, Hoekstra-Weebers JEHM (2007). Burnout in medical residents: a review. Med Educ.

[16] Mougalian SS, Lessen DS, Levine RL, Panagopoulos G, Von Roenn JH, Arnold RM (2013). Palliative care training and associations with burnout in oncology fellows. J Support Oncol.

[17] Prins JT, Hoekstra-Weebers JEHM, Gazendam-Donofrio SM, Dillingh GS, Bakker AB, Huisman M (2010). Burnout and engagement among resident doctors in the Netherlands: a national study. Med Educ.

[18] Sood A, Prasad K, Schroeder D, Varkey P (2011). Stress management and resilience training among Department of Medicine Faculty: a pilot randomized clinical trial. J Gen Int Med.

[19] Loprinzi CE, Prasad K, Schroeder DR, Sood A (2011). Stress Management and Resilience Training (SMART) Program to decrease stress and enhance resilience among breast cancer survivors: a pilot randomized clinical trial. Clin Breast Cancer.

[20] Sood A, Sharma V, Schroeder DR, Gorman B (2014). Stress Management and Resiliency Training (SMART) Program among Department of Radiology Faculty: a pilot randomized clinical trial. EXPLORE: J Sci Heal.

[21] McCullough ME, Emmons RA, Tsang JA (2002). The grateful disposition: a conceptual and empirical topography. J Pers Soc Psychol.

[22] Diener E, Emmons RA, Larsen RJ, Griffin S (1985). The Satisfaction with Life Scale. J Pers Asses.

[23] Lyubomirsky S, Lepper H (1999). A measure of subjective happiness: preliminary reliability and construct validation. Soc Indic Res.

[24] Maslach C, Jackson SE (1981). The measurement of experienced burnout. J Org Behav.

[25] Cohen S, Kamarck T, Mermelstein R (1983). A global measure of perceived stress. J Health Soc Behav.

[26] West CP, Dyrbye LN, Satele DV, Sloan JA, Shanafelt TD (2012). Concurrent validity of single-item measures of emotional exhaustion and depersonalization in burnout assessment. J Gen Int Med.

[27] Dyrbye L, Sotile W, Boone S, West C, Tan L, Satele D (2013). A survey of U.S. physicians and their partners regarding the impact of work–home conflict. J Gen Int Med.

[28] Andreou E, Alexopoulos EC, Lionis C, Varvogli L, Gnardellis C, Chrousos GP (2011). Perceived stress scale: reliability and validity study in Greece. Int J of Environ Res Public Health.

[29] Guntupalli KK, Fromm RE (1996). Burnout in the internist-intensivist. Intensive Care Med.

